# Biodegradable, Flexible, and Transparent Conducting Silver Nanowires/Polylactide Film with High Performance for Optoelectronic Devices

**DOI:** 10.3390/polym12030604

**Published:** 2020-03-06

**Authors:** Junjun Wang, Junsheng Yu, Dongyu Bai, Zhuobin Li, Huili Liu, Ying Li, Shanyong Chen, Jiang Cheng, Lu Li

**Affiliations:** 1 State Key Laboratory of Electronic Thin Films and Integrated Devices, School of Optoelectronic Science and Engineering, University of Electronic Science and Technology of China (UESTC), Chengdu 610054, China; 2 Chongqing Key Laboratory of Materials Surface & Interface Science, School of Materials Science and Engineering, Chongqing University of Arts and Sciences, Chongqing 402160, China; 3 Chongqing Key Laboratory of Environmental Materials and Remediation Technologies, Chongqing University of Arts and Sciences, Chongqing 402160, China

**Keywords:** polylactide, stereocomplex, silver nanowires, green optoelectronic devices

## Abstract

As a synthetic renewable and biodegradable material, the application of polylactide (PLA) in the green flexible electronics has attracted intensive attention due to the increasingly serious issue of electronic waste. Unfortunately, the development of PLA-based optoelectronic devices is greatly hindered by the poor heat resistance and mechanical property of PLA. To overcome these limitations, herein, we report a facile and promising route to fabricate silver nanowires/PLA (AgNW/PLA) film with largely improved properties by utilizing the stereocomplex (SC) crystallization between poly(L-lactide) (PLLA) and poly(D-lactide) (PDLA). Through embedding the AgNW networks into the PLLA:PDLA blend matrix via a transfer method, the AgNW/PLLA:PDLA film with both high transparency and excellent conductivity was obtained. Compared with the AgNW/PLLA film, the formation of SC crystallites in the composites matrix could significantly enhance not only heat resistance but also mechanical strength of the AgNW/PLLA:PDLA film. Exceptionally, the AgNW/PLLA:PDLA film exhibited superior flexibility and could maintain excellent electrical conductivity stability even under the condition of 10,000 repeated bending cycles and 100 tape test cycles. In addition, the organic light-emitting diodes (OLEDs) with the AgNW/PLLA:PDLA films as electrodes were successfully fabricated in this work for the first time and they exhibited highly flexible, luminous, as well as hydrolytic degradation properties. This work could provide a low-cost and environment-friendly avenue towards fabricating high-performanced PLA-based biodegradable electronics.

## 1. Introduction

With the advent of the Internet of Things era, diverse electronics including sensors, displays, and antennas have attracted a great deal of attention in recent years [1,2]. However, human beings are deeply troubled by electronic waste (e-waste) while they are enjoying the convenience of electronics [3,4]. So far, the majority of e-waste is handled by landfill and incineration, but these two treatment methods may lead the toxic and hazardous components of e-waste to go directly into the soil, water, and air, finally resulting in serious ecological pollution [5]. In this case, developing biodegradable and recyclable electronics has become a particularly important issue [6].

The substrate of electronics occupies the main volume and weight and thus generates more waste than the functional layers deposited on it [7]. If the conventional nondegradable substrates (e.g., silicon, glass, and petroleum-based plastics) are replaced by biodegradable materials, the e-waste problem will be greatly alleviated. Nowadays, many natural renewable polymer materials such as cellulose, silk, chitin, and so on [8,9] have been applied as the substrate of various electronics (sensors [10,11,12], solar cells [13,14], transistors [15,16], and printed circuits [17,18]) owing to their apparent advantages of biodegradability, lightweight, and flexibility. Nevertheless, these natural polymer materials suffer from the drawbacks of high surface roughness, low mechanical strength, poor durability, inferior thermal resistance, bad shape stability in solution, and complex processing [19,20,21]. For example, although the porous nature and high roughness of cellulose nanofibers are beneficial to electrochemical applications (e.g., supercapacitors) because the increasement of specific surface area can allow efficient and rapid mass transport [22,23,24], it is not suitable for solar cells, organic light-emitting diodes (OLEDs), and printed electronics which require a smooth and non-porous substrate to prevent cracks, breaks, and shunts in the films [25]. Silk is very vulnerable to insect damage and denatured by high temperature, acid, and alkali [13,26,27]. Chitin endures the defects of insolubility and recalcitrance due to the supramolecular crystalline nanofibers [28,29]. Generally, the limitations of natural renewable polymers mentioned above significantly impede their applications in preparing high-performance electronics [7].

By contrast, the synthetic biodegradable polymer polylactide (PLA) represents the most promising alternative to replace the nondegradable substrates of electronics, because it possesses not only absolute renewability from starch-containing crops and full biodegradability in soil after use, but also low cost, easy processability, excellent transparency, superior resistivity to chemicals, and better mechanical performance compared to other synthetic biopolymers [30]. Due to the excellent physical properties and various advantages, the application of PLA in the green electronics has attracted considerable research interest in recent years. For instance, the highly conductive and flexible silver nanowires/PLA (AgNW/PLA) composites have been fabricated by solution mixing [31], transferring [32], and electrospinning [33], and they exhibit great application potential in flexible electronics and optoelectronics fields. However, up to now, the large-scale use of PLA in electronic devices still faces some obstacles mostly related to its unsatisfactory properties like inferior mechanical strength and poor heat resistance. Recently, stereocomplex (SC) crystallization has been reported to occur readily upon blending poly(L-lactide) (PLLA) with its enantiomer poly(D-lactide) (PDLA), and it has been demonstrated that the in situ formed SC crystallites possess a much higher melting temperature (220–230 °C) than that of homocrystallites of PLLA or PDLA (160–175 °C) as well as superior physicomechanical properties such as excellent heat/chemical resistance [34,35], hydrolysis/heat stability [36,37], and mechanical performance [38]. Inspired by these fascinating results, in this work, we proposed a new interesting strategy to improve the properties of AgNW/PLA materials by taking advantage of SC crystallites. Through embedding the AgNW networks into the PLLA:PDLA blend matrix via a transfer method [39,40], a transparent and flexible AgNW/PLLA:PDLA film was fabricated. This AgNW/PLLA:PDLA film had excellent conductivity and low surface roughness. More importantly, compared with the AgNW/PLLA film, the AgNW/PLLA:PDLA film showed better thermal resistance, mechanical strength, and hydrolysis resistance owing to the formation of SC crystallites. Finally, a flexible and biodegradable OLED device based on the AgNW/PLLA:PDLA film was successfully fabricated for the first time, directly indicating the great potential of this SC crystallites-mediated biodegradable material in the application of optoelectronic devices.

## 2. Experimental Section

### 2.1. Materials

Commercial PLLA (grade 4032D) was purchased from Nature Works Co. LLC (Blair, NE, USA), with a weight-average molecular weight (*M_w_*) of 170 kg/mol, and a polydispersity (PDI) of 1.74. PDLA was obtained from Changchun Sino Biomaterials Co., Ltd., (Changchun, China) with a *M_w_* of 167 kg/mol and a PDI of 1.58. AgNWs were supplied by Zhejiang Kechuang Advanced Materials Co., Ltd., (Hangzhou, China), with an average wire diameter of 25–35 nm and length of 10–20 μm. Chloroform, Sodium hydroxide and isopropyl alcohol were purchased from Chengdu Kelong Chemical Reagent Factory (Chengdu, China) and were used as received. 

### 2.2. Sample Preparation

#### 2.2.1. Fabrication of the AgNW/PLLA:PDLA Film

**Figure 1:**
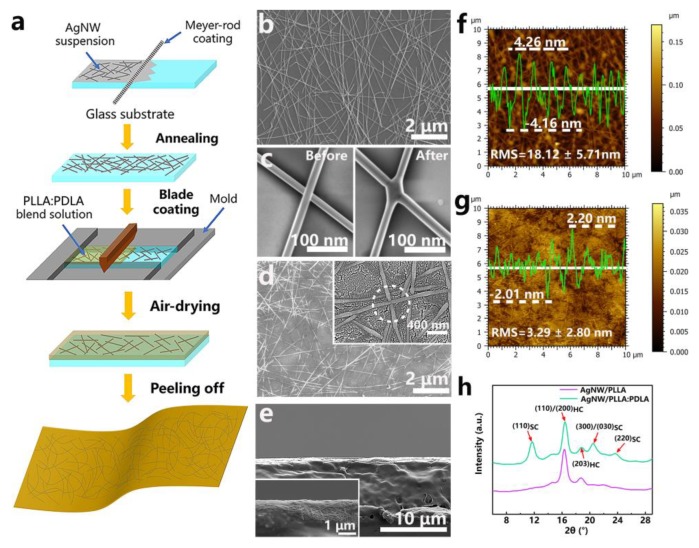
(**a**) Schematic illustration of the fabrication process of the AgNW/PLLA:PDLA film. SEM images of (**b**) the surface of the AgNW/Glass film with a 72 mg/m^2^ AgNW area densities, (**c**) the adjacent AgNWs of the AgNW/Glass film before and after annealing treatment, and (**d**) the surface of the AgNW/PLLA:PDLA film with a 72 mg/m^2^ AgNW area density. (**e**) Cross-sectional SEM images of the AgNW/PLLA:PDLA film with a 72 mg/m^2^ AgNW area density. AFM images (the inserted green curve is the surface height of the marked white line) of (**f**) the AgNW/Glass film and (**g**) the AgNW/PLLA:PDLA film with a 72 mg/m^2^ AgNW area density. (**h**) WAXD profiles of the AgNW/PLLA and AgNW/PLLA:PDLA films.

Figure 1a presents the schematic illustration of the fabrication procedure of the AgNW/PLLA:PDLA film. Initially, the AgNW dispersion (0.1 wt% in isopropyl alcohol) was dropped on the one end of the glass slide which was placed flat on the drawdown machine (DP-8301, Gardco, Pompano Beach, FL, USA) and then a Meyer rod (RSD 20, Gardco, Pompano Beach, FL, USA) was used instantly to spread the dispersion over the glass surface uniformly. In order to obtain a uniform AgNW thin film and decrease the resistance of the AgNW percolation networks, the AgNW/Glass was annealed on a hot plate at 100 °C for 10 min, soaked in deionized (DI) water for 10 min, and subsequently annealed at 130 °C for another 8 min. The transparency and conductivity of the film were dependent on the AgNW area density (defined by the deposited weight per unit area) which could be controlled by the coating times. Subsequently, PLLA and PDLA (PLLA/PDLA = 1:1, wt/wt) were separately dissolved in chloroform (50 g·L^−1^) at room temperature, and PLLA:PDLA blend solution was obtained by mixing the prepared PLLA and PDLA solution with vigorous stirring. After then, the PLLA:PDLA blend solution was blade-coated on the AgNW/Glass (glass slide coated with AgNW networks facing up). Finally, the AgNW/PLLA:PDLA film was peeled off after air-drying for 12 h and drying at 60 °C in vacuum oven for 4 h. As comparison, the AgNW/PLLA film was fabricated under the same condition.

#### 2.2.2. Preparation of the AgNW/PLLA:PDLA Film-based OLEDs 

The as-prepared AgNW/PLLA:PDLA film was cleaned by DI water and then dried by clean nitrogen. The structure of the fabricated OLEDs included hole-transport layer, emitting layer, electron transport layer, and cathode which were all deposited on the AgNW/PLLA:PDLA film layer by layer. The specific materials and thicknesses were as followed: AgNW/PLLA:PDLA (20 μm)/HAT-CN (15 nm)/TAPC (40 nm)/TPX-ZPO (15 nm)/Ir(bt)_2_acac (0.5 nm)/B4PYMPM (40 nm)/LiF (1 nm)/Al (100 nm).

### 2.3. Characterizations 

#### 2.3.1. Scanning Electron Microscopy (SEM)

All the scanning electron microscopy measurements were carried out on a field emission SEM (GeminiSEM 300, ZEISS, Jena, Germany) at an accelerating voltage of 3 kV. Specimens used for the cross-sectional observation were prepared by cryo-fracturing the film in liquid nitrogen. A thin layer of gold was coated both on the surface and cross-section of the films before observations.

#### 2.3.2. Atomic Force Microscopy (AFM)

The surface roughness and topography of the films were evaluated using atomic force microscopy (AFM 5500, Agilent, Santa Clara, CA, USA) in tapping mode. The scan area was 10 μm × 10 μm.

#### 2.3.3. Wide-angle X-ray Diffraction (WAXD)

WAXD patterns were recorded on an automatic X-ray diffractometer (TD-3500, Dandong, China) equipped with a Cu Kα radiation source (λ = 0.154 nm, 30 kV, 20 mA). The specimens were scanned in the diffraction angle (2θ) range of 5°–30° at a scanning speed of 5°/min. 

#### 2.3.4. Optical Transmittance Spectra 

The optical transmittance measurements were conducted on a UV-visible spectrophotometer (Cary 5000, Agilent, Santa Clara, CA, USA) in the wavelength range of 300–800 nm with air as the reference. 

#### 2.3.5. Sheet Resistance

The sheet resistances were measured by a four-point probe (RTS-5, Guangzhou Four-Point Probe, Guangzhou, China). For each sample, the reported resistance value was averaged from at least five independent specimens.

#### 2.3.6. Differential Scanning Calorimetry (DSC) and Heat Resistance Test

The melting behaviors were analyzed by DSC measurements (Q2000, TA, New Castle, DE, USA) under nitrogen atmosphere. For each measurement, about 5 mg specimen was sealed in an aluminum pan and then heated from 30 °C to 250 °C at a heating rate of 10 °C/min. The real-time sheet resistance of each film sample after annealing at different temperatures for 30 min was measured to evaluate the heat resistance of the films.

#### 2.3.7. Mechanical Test

The tensile test was carried out at room temperature by using a dynamic mechanical testing machine (TestStar, WANCE, Shenzhen, China) with a crosshead speed of 5 mm/min. The size of tensile bars is 15 mm × 4 mm × 0.02 mm (length × width × thickness) and the reported data for each sample was averaged from at least six independent specimens.

The bending test was performed on a dynamic mechanical testing machine (TestStar, WANCE, Shenzhen, China). Both ends of the film were firmly fixed to the two crossheads. The bending radius was 3 mm and bending cycles ranged from 0 to 10,000.

The adhesion strength test was conducted by 13-mm-wide piece of 3M scotch tape (3M, St. Paul, MN, USA). After attaching the test area of the film surface completely, the tape was removed rapidly. The test cycles ranged from 0 to 1000.

#### 2.3.8. Measurements of the OLEDs

The luminance-voltage-current density curves of the OLEDs were measured by a source meter (Keithley 2400, Tektronix, Beaverton, OR, USA) and a calibrated silicon photodetector. When measuring the luminance of the OLEDs, the OLEDs were attached to the photodetector to receive as much light flux as possible. The active area was 10 mm^2^ which was defined by the overlap of the anode and cathode.

#### 2.3.9. Hydrolytic Degradation Test 

The hydrolytic degradation of the discarded OLEDs (15 mm × 15 mm × 20 μm) was carried out in sodium hydroxide (NaOH) solution (pH = 13) at 37 °C. At the predetermined time intervals (i.e., 12 h), the specimen was taken from the solution, washed with DI water, and then dried in vacuum at 50 °C to get a constant weight. The degradation of the sample was determined by the variation of weight loss as a function of degradation time. At least five specimens were used to obtain the average weight loss values.

## 3. Results and Discussion

### 3.1. Fabrication and Structure of the AgNW/PLLA:PDLA Film

The fabrication process of the AgNW/PLLA:PDLA film is schematically illustrated in Figure 1a. The AgNW/PLLA:PDLA film was successfully obtained via transferring the AgNW networks into the matrix of the PLLA:PDLA blend film. Figure 1b shows the SEM image of AgNW networks on glass substrate. AgNWs were distributed randomly with no obvious aggregation and connected to each other forming well networks. To decrease the resistance of the percolation network, the AgNW/Glass film was annealed at the temperature of 100 °C and 130 °C. It is demonstrated in Figure 1c that the annealing treatment can result in the fusion of adjacent AgNWs because of the nanowelding effect. The higher magnification SEM image inset in Figure 1d exhibits that nanowires in the AgNW/PLLA:PDLA film were embedded in polymer matrix due to the PLLA:PDLA blend solution filling the pores between the nanowires [39] and the existence of fused nanowires indicated the unaltered well connection of AgNW networks after transferring. The cross section of AgNW/PLLA:PDLA film in Figure 1e manifests again that the thin conductive layer of AgNW with well networks was embedded into the matrix of the PLLA:PDLA film. In addition, as shown in Figure 1f,g, the AgNW/PLLA:PDLA film possessed a lower surface roughness (RMS, 3.29 ± 2.80 nm) than that of the AgNW/Glass film (RMS, 18.12 ± 5.71 nm) which indicated that the AgNW/PLLA:PDLA film is suitable for the fabrication of high-performance optoelectronic devices. WAXD measurements were conducted to demonstrate the formation of SC crystallites in the AgNW/PLLA:PDLA film. As shown in Figure 1g, the WAXD pattern of the AgNW/PLLA film exhibited two characteristic diffraction peaks at about 16.4° and 18.8°, corresponding to the (110)/(200), and (203) planes of homocrystallites. Besides the two peaks, the other three peaks at around 11.7°, 20.5°, and 23.7° representing the (110), (300)/(030), and (220) planes of SC crystallites [41,42], respectively, appeared in the AgNW/PLLA:PDLA film, which indicated the simultaneous formation of homocrystallites and SC crystallites in the AgNW/PLLA:PDLA film.

### 3.2. Optical Transmittance and Sheet Resistance of the AgNW/PLLA:PDLA Film

**Figure 2:**
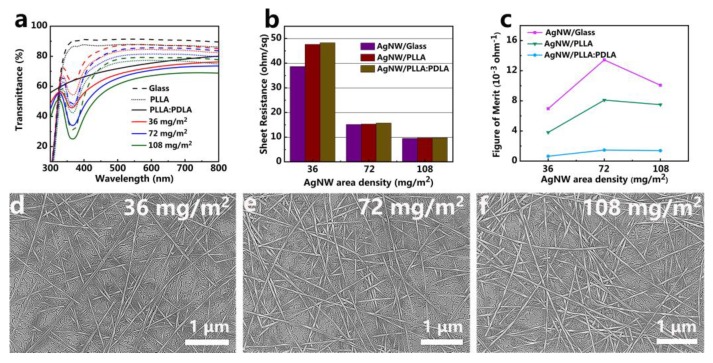
(**a**) Optical transmittance, (**b**) sheet resistance, and (**c**) figure of merit of the AgNW/Glass, AgNW/PLLA, and AgNW/PLLA:PDLA films with different AgNW area densities. (**d**–**f**) The corresponding SEM images of the surface of the AgNW/PLLA:PDLA film with different AgNW area densities. The same color lines mean the same AgNW area density in figure (**a**).

As a transparent and conductive film, optical transmittance and sheet resistance (*R_S_*) are two critical parameters to evaluate its quality and practical applications for optoelectronic devices. Figure 2a presents the optical transmittances of the AgNW film with different AgNW area densities (36 mg/m^2^, 72 mg/m^2^, and 108 mg/m^2^) deposited on glass or transferred by PLLA and PLLA:PDLA blend solution. Obviously, for the same substrate, the optical transmittance of the film decreased with increasing the AgNW area density due to the fact that more nanowires on the substrate can cause more light to be reflected and absorbed [43,44]. Compared with the AgNW/Glass film, the optical transmittance of the AgNW/PLLA and AgNW/PLLA:PDLA film with the same AgNW area density dropped about 2%–4% and 10%–20% owing to the crystallization of the polymer matrix, respectively. The variation in *R_S_* with different AgNW area densities before and after transferring is shown in Figure 2b. With AgNW area density increasing from 36 to 108 mg/m^2^, the *R_S_* decreased significantly from 38.7 ohm/sq (Glass), 47.6 ohm/sq (PLLA), and 48.3 ohm/sq (PLLA:PDLA) to 9.5 ohm/sq (Glass) and 9.7 ohm/sq (PLLA, PLLA:PDLA) due to the formation of more electronic transmission channels [45]. The reduction of the gap between the sheet resistance of the AgNW/Glass and the AgNW/PLLA:PDLA indicated that nanowires were transferred more and more thoroughly.

The figure of merit (FoM, *Φ_TC_*) based on *R_S_* and transmittance was calculated to determine the optimum area density of AgNW on glass or embedded into PLLA:PDLA layer in the following Equation (1) [46]:

(1) $$\Phi_{TC}=\frac{T^{10}}{R_{S}}$$

where *T* is the transmittance at a wavelength of 550 nm. Figure 2c exhibits the calculated *Φ_TC_* as a function of the AgNW area density. For the AgNW/Glass film, the *Φ_TC_* values increased from 7.0 to 13.4 and then decreased to 10.1 with increasement of the AgNW area density from 36 to 108 mg/m^2^. For the AgNW/PLLA and AgNW/PLLA:PDLA films, they had the same trend and the optimal *Φ_TC_* value was 8.1 and 1.5 (72 mg/m^2^), respectively. The distinct decline in *Φ_TC_* values after transferring mainly arose from the loss of optical transmittance while the sheet resistance was nearly unchanged. Table 1 gives the comparison of optical transmittance, sheet resistance, and figure of merit of the AgNW/Glass, AgNW/PLLA and AgNW/PLLA:PDLA films at different AgNW area densities. All of the AgNW films covered on glass, PLLA, and PLLA:PDLA showed the highest *Φ_TC_* value when the area density was 72 mg/m^2^. As shown in Figure 2d,f, with the increasement in the AgNW area density, the AgNW networks became denser and more nanowires were connected together, which resulted in the lower transmittance and better conductivity of the film, respectively.

### 3.3. Melting Behaviors and Heat Resistance of the AgNW/PLLA:PDLA Film

**Figure 3:**
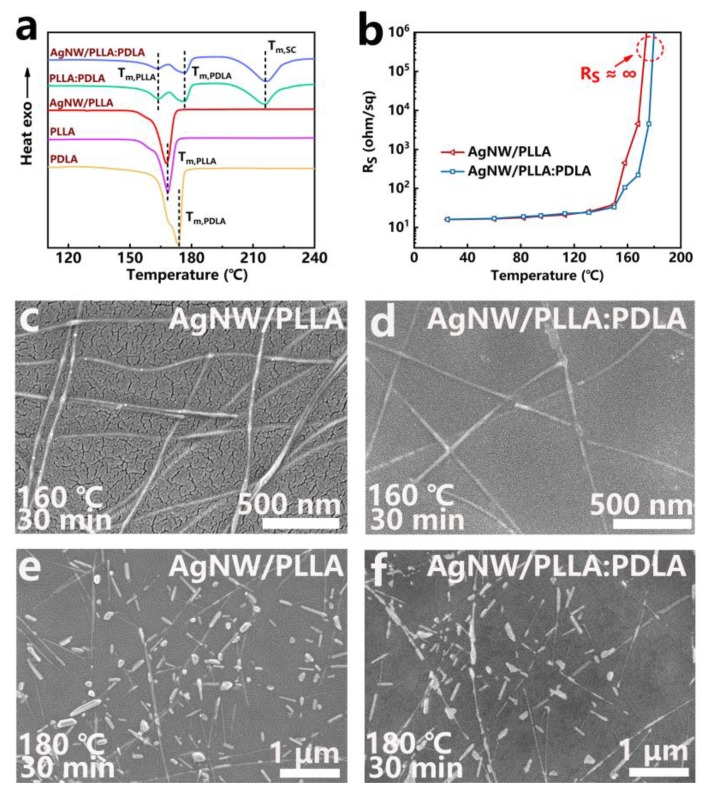
(**a**) DSC measurements of the PDLA, PLLA, AgNW/PLLA, PLLA:PDLA, and AgNW/PLLA:PDLA films. (**b**) Variations of the *R_S_* of the AgNW/PLLA and AgNW/PLLA:PDLA films as a function of the annealing temperature (the annealing time is 30 min). (**c**–**f**) SEM images of the cooled down AgNW/PLLA and AgNW/PLLA:PDLA films after annealing at 160 °C or 180 °C for 30 min.

DSC measurements were conducted to explore the melting behaviors of the AgNW/PLLA:PDLA film. As shown in Figure 3a, the melting temperatures of the PDLA film (*T_m,PDLA_*) and the PLLA film (*T_m,PLLA_*) appeared at around 174 °C and 168 °C, respectively. However, except *T_m,PDLA_* and *T_m,PLLA_*, another new melting temperature ascribed to the SC crystallites was observed at around 217 °C in the melting curves of the PLLA:PDLA and AgNW/PLLA:PDLA films, which further verified the existence of SC crystallites in the AgNW/PLLA:PDLA film. It should be noted that the addition of AgNWs had little effect on the melting temperatures of the films. The variations of the *R_S_* of the AgNW/PLLA and AgNW/PLLA:PDLA films were recorded as a function of the annealing temperatures (Figure 3b). The AgNW/PLLA and AgNW/PLLA:PDLA films shared a similar trend in *R_S_* when the annealing temperature was below 160 °C. Nevertheless, the *R_S_* of the AgNW/PLLA film exhibited a sharp growth (from less than 50 ohm/sq to over 400 ohm/sq) at about 160 °C, while the *R_S_* of the AgNW/PLLA:PDLA film was still around 100 ohm/sq. Figure 3c is the SEM image of the cooled down AgNW/PLLA film after annealing at 160 °C for 30 min, where the nanowires are observed to remain intact in PLLA matrix. Actually, the *R_S_* of the cooled down AgNW/PLLA film after annealing at 160 °C for 30 min was about 35 ohm/sq, far less than 400 ohm/sq. This big difference clearly indicated that the melting of PLLA matrix at 160 °C can greatly destroy the conductive networks of AgNW (the reason of the sharp growth of *R_S_*). By contrast, the much higher *T_m,SC_* of SC crystallites can endow the AgNW/PLLA:PDLA film with better heat resistance and, hence, the AgNW/PLLA:PDLA film can still keep a good conductive network even after annealing at 160 °C for 30 min (Figure 3d). As the annealing temperature grew to 180 °C, the *R_S_* of the AgNW/PLLA and AgNW/PLLA:PDLA films both approached infinity. It can be seen in Figure 3e,f that almost all nanowires transformed into isolated droplets when annealing at 180 °C for 30 min. For the AgNW/PLLA film, both the complete melting of the PLLA matrix and the breakdown of nanowires caused the AgNW/PLLA film to become non-conducting at 180 °C. While for the AgNW/PLLA:PDLA film, the breakdown of nanowires was the main reason for the increase in the *R_S_* of the AgNW/PLLA:PDLA film at 180 °C. 

### 3.4. Mechanical Properties of the AgNW/PLLA:PDLA Film

**Figure 4:**
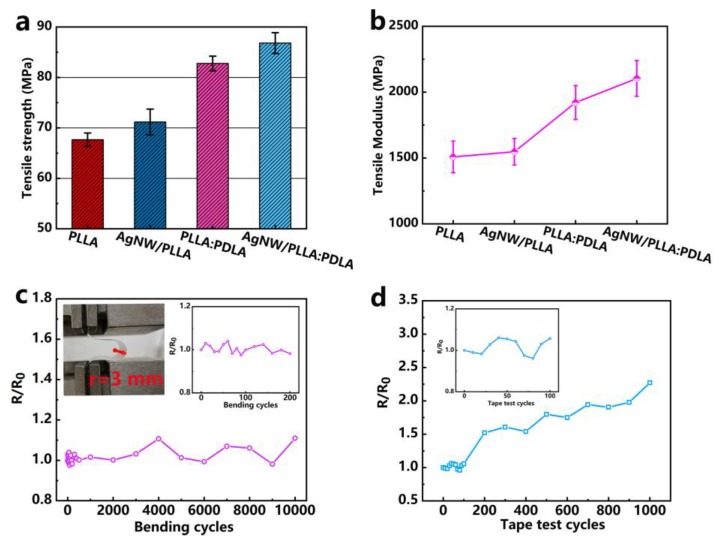
(**a**) Tensile strength and (**b**) tensile modulus of the PLLA, AgNW/PLLA, PLLA:PDLA, and AgNW/PLLA:PDLA films. (**c**) The bent state of the film with the radius of 3 mm, *R/R_0_* of the AgNW/PLLA:PDLA film during bending test. (**d**) *R/R_0_* of the AgNW/PLLA:PDLA film at different adhesion testing cycles with 3M scotch tape. *R* is the real-time sheet resistance and *R_0_* is the initial sheet resistance of the AgNW/PLLA:PDLA film.

The tensile strength and modulus of the films are shown in Figure 4a,b. Obviously, the incorporation of AgNWs into the PLLA and PLLA:PDLA blend matrix can simultaneously enhance their tensile strength and modulus. Besides, a much more rapid increasement in the tensile strength and modulus was observed in the PLLA:PDLA film and AgNW/PLLA:PDLA films where SC crystallites were formed in the polymer matrix, distinctly demonstrating the significant role of SC crystallites in strengthening the PLA materials [47]. For foldable and wearable devices, flexibility and sheet resistance stability are two essential parameters [48]. Herein, cyclic bending test with a bending radius of 3 mm (see Figure 4c) was conducted to simultaneously explore the flexibility and the sheet resistance stability of the AgNW/PLLA:PDLA film. As shown in Figure 4c, the *R/R_0_* value of the AgNW/PLLA:PDLA film always fluctuated around 1.0 during the 10,000 cyclic bending cycles, confirming the excellent sheet resistance stability and commendable flexibility of the AgNW/PLLA:PDLA film. Generally, the AgNW film is fabricated by directly depositing nanowires on the substrate. In this case, the weak adhesion force between AgNW and substrate usually leads the AgNW to be easily detached by tape or other friction [39,44]. Therefore, to verify the adhesion force between AgNW networks and PLLA:PDLA film, a 3M tape test was performed on the surface of the AgNW/PLLA:PDLA film. It can be seen in Figure 4d that the *R/R_0_* of the AgNW/PLLA:PDLA film changed slightly in the testing range of 100 cycles which indicated that the AgNW/PLLA:PDLA film presented good adhesion between AgNW networks and PLLA:PDLA film, and then the *R_S_* started to rise up with the tape test cycles increasing due to the peeling of the AgNWs by the tape. However, compared to the AgNW film on glass, the AgNW/PLLA:PDLA film presents good adhesion overall due to nanowires embedded into the surface of PLLA/PDLA layer.

### 3.5. Application of the AgNW/PLLA:PDLA Film in Optoelectronic Devices

**Figure 5:**
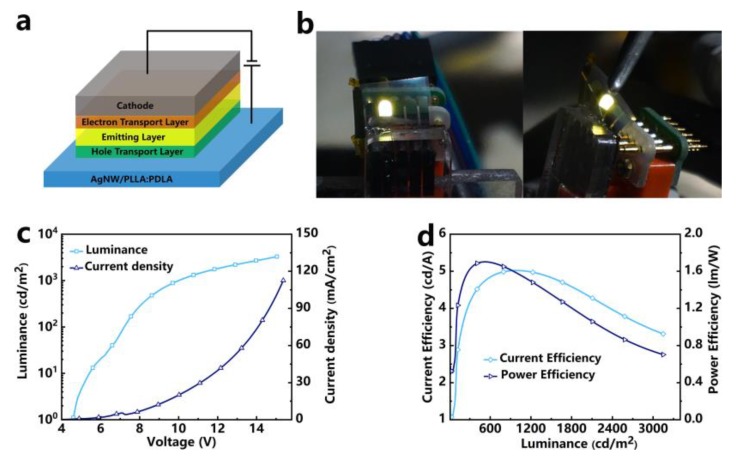
(**a**) Schematic illustration of the OLEDs structure and (**b**) photographs of turned-on OLEDs based on the AgNW/PLLA:PDLA film. (**c**) Luminance-voltage-current density curves and (**d**) current efficiency-luminance-power efficiency curves of the OLEDs.

In this work, the AgNW/PLLA:PDLA film with a 72 mg/m^2^ area density was used as the substrate and electrode to fabricate optoelectronic devices. The schematic structure of the OLEDs is illustrated in Figure 5a which included electrode, hole-transport layer, emitting layer, electron transport layer, and cathode which layer by layer as followed: AgNW/PLLA/PDLA (20 μm)/HAT-CN (15 nm)/TAPC (40 nm)/TPX-ZPO (15 nm)/Ir(bt)_2_acac (0.5 nm)/B4PYMPM (40 nm)/LiF (1 nm)/Al (100 nm).

Figure 5b shows the optical image of the OLEDs. It was lit when the voltage was applied and could remain luminous when it was bent, clearly indicating the excellent conductivity and superior flexibility of the AgNW/PLLA:PDLA film-based OLEDs. The turn-on voltage (defined at 1 cd/m^2^), maximum luminance and external quantum efficiency (EQE) of the OLEDs were 4.6 V, 3283 cd/m^2^ (at 15.05 V) and 1.51%, respectively (Figure 5c). As shown in Figure 5d, the current efficiency (CE) and power efficiency (PE) reached 5.02 cd/A (luminance of 967 cd/m^2^) and 1.70 lm/w (luminance of 516 cd/m^2^), respectively. It should be noted that the PE ($\eta_{p}$) of the OLEDs was calculated by the following Equation (2):

(2) $$\eta_{p}=\frac{\pi AL}{I_{OLED}V}$$

where *A* is the active area, *L* is the measured luminance of the OLEDs, $I_{OLED}$ is the current of the OLEDs at the applied voltage, and *V* is the applied voltage. When the *V* was applied, the $I_{OLED}$ can be acquired by the source meter. As *π* and *A* are constants, it is only needed to accurately measure the luminance at each voltage by a calibrated silicon photodetector. To eliminate the effect of the angular distribution of light on calculating the PE of the OLEDs, the OLEDs were attached to the photodetector to receive as much light flux as possible when measuring the luminance of the OLEDs. Therefore, the light emitted by the OLEDs can be seen as being vertically irradiated onto the photodetector without being diverted to other places.

The performance of the OLEDs based on the AgNW/PLLA:PDLA film can be further improved by optimizing the structure, materials, and process parameters of the OLEDs in future work.

### 3.6. Hydrolytic Degradation Property of the AgNW/PLLA:PDLA-based OLEDs

**Figure 6:**
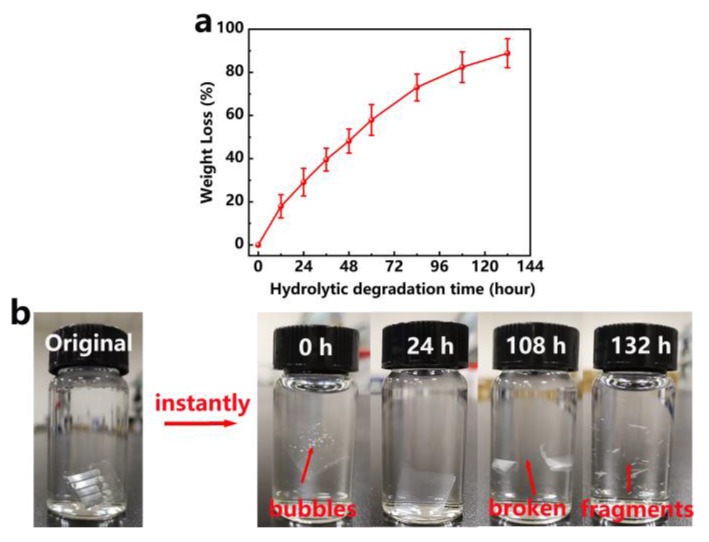
(**a**) Hydrolytic degradation rate of the OLEDs based on the AgNW/PLLA:PDLA film. (**b**) Photographs of the OLEDs immersed into NaOH solution for different hours.

The hydrolysis of PLA is actually the hydrolysis of ester groups. The hydrolysis process is carried out under the catalysis of hydrogen ions and hydroxide ions, and the high concentration of hydroxide ions in the alkaline medium will strongly promote the hydrolysis of the ester group of the PLA-based material toward the positive reaction direction [49]. The degradation property of the OLEDs based on the AgNW/PLLA:PDLA film was carried out by the hydrolytic degradation in NaOH solution test. Figure 6a exhibits that the weight loss of OLEDs increased with the hydrolytic degradation time and can reach up to about 91% after hydrolytic degradation for 132 h. During the hydrolytic degradation test (see Figure 6b), several bubbles upon the device can be clearly observed when the specimen was immersed into the solution which was ascribed to the reaction between aluminum (cathode of the OLEDs) and the NaOH solution forming hydrogen gas. The OLEDs was broken after hydrolytic degradation in NaOH solution for 108 h and further broken down into numerous fragments after 132 h, suggesting that the OLEDs based on the AgNW/PLLA:PDLA film possessed good biodegradable properties.

## 4. Conclusions

In summary, we have demonstrated a feasible strategy to fabricate the highly flexible and transparent conductive AgNW/PLLA:PDLA film with largely improved properties. The AgNW/PLLA:PDLA film was comprised of AgNWs embedded into PLLA:PDLA matrix, and possessed low surface roughness and stable electrical conductivity even under the conditions of 10,000 bending or 100 tape test cycles. More importantly, the AgNW/PLLA:PDLA film exhibited superior tensile strength, tensile modulus and heat resistance owing to the in situ formation of SC crystallites in the polymer matrix. As proof of viability, the optimal AgNW/PLLA:PDLA film (*T* ≈ 68.6%, *Rs* ≈ 15.7 ohm/sq) was chosen as the electrode for the OLEDs and the as-prepared OLEDs exhibited highly flexible, luminous, and biodegradable properties. Overall, the high-performanced AgNW/PLLA:PDLA film can endow itself with more potential applications in green disposable electronics which is beneficial to human sustainable development.

## Figures and Tables

**Table 1:** Comparison of optical transmittance, sheet resistance, and figure of merit of the AgNW/Glass, AgNW/PLLA, and AgNW/PLLA:PDLA films at different AgNW area densities.

Substrate	AgNW Area Density (mg/m^2^)	Transmittance at 550 nm (%)	Sheet Resistance (ohm/sq)	Figure of Merit (10^−3^ ohm^−1^)
	36	87.7	38.7	7.0
Glass	72	85.3	15.2	13.4
	108	79.1	9.5	10.1
	36	84.3	47.6	3.8
PLLA	72	81.2	15.3	8.1
	108	76.9	9.7	7.5
	36	70.7	48.3	0.6
PLLA:PDLA	72	68.6	15.7	1.5
	108	65.1	9.7	1.4
